# Sudden unexpected death after initial infusion of rituximab for Waldenström macroglobulinemia/lymphoplasmacytic lymphoma: an autopsy case

**DOI:** 10.1186/s13000-024-01519-9

**Published:** 2024-06-28

**Authors:** Shojiro Ichimata, Yukiko Hata, Kazuhiro Nomoto, Tsutomu Sato, Naoki Nishida

**Affiliations:** 1 Department of Legal Medicine, Faculty of Medicine, University of Toyama, 2630 Sugitani, Toyama, 930-0194 Japan; 2 Department of Pathology, Koseiren Takaoka Hospital, Takaoka, Japan; 3 Department of Hematology, Toyama University Hospital, Toyama, Japan

**Keywords:** Amyloid immunoglobulin light chain amyloidosis, Infusion-related reactions, Rituximab, Sudden unexpected death, Waldenström’s macroglobulinemia/Lymphoplasmacytic lymphoma

## Abstract

**Background:**

Waldenström’s macroglobulinemia (WM) is defined as a lymphoplasmacytic lymphoma (LPL) involving the bone marrow (BM) with presence of IgM monoclonal protein, and comprises > 95% of all LPL cases. Rituximab-based regimens have been predominant in the management of WM. Infusion-related reactions (IRRs) are a primary concern with rituximab, although it is generally better tolerated with less toxicity than conventional anticancer agents. Here, we present an autopsy case of an elderly man who died suddenly after receiving the initial infusion of rituximab for WM/LPL.

**Case presentation:**

An 84-year-old man was found dead in his bedroom. He had undergone the initial intravenous rituximab infusion for progressive anemia related to Waldenström’s macroglobulinemia/lymphoplasmacytic lymphoma (WM/LPL) approximately 15 h before death. Although the protocol for rituximab administration and additional medication was considered appropriate, he exhibited several symptoms consistent with infusion-related reactions (IRRs) during the infusion. Autopsy revealed monotonous proliferation of small-to-medium-sized lymphocytic cells in the bone marrow, consistent with the premortem diagnosis of WM/LPL. Additionally, immunoglobulin λ-light chain-derived amyloid (ALλ) deposition was identified in all organs other than the brain. Although ALλ deposition and LPL infiltration were found in the heart, they were not severe enough to cause severe functional impairment. Severe congestion and/or edema were observed in the lungs, liver, and brain. Although significant inflammatory cell infiltration was not found in any organs, laboratory tests revealed elevated serum levels of inflammatory cytokines, including interleukin-1β, interleukin-6, tumor necrosis factor-α and the presence of IgM-λ monoclonal protein.

**Conclusion:**

Acute IRRs associated with the initial rituximab infusion were the major contributing factor to his sudden unexpected death. The autopsy findings of present case suggest the necessity for thorough monitoring of older patients with WM/LPL undergoing rituximab treatment, particularly when pronounced IRRs occur during the first administration, in addition to investigating complications of WM/LPL before infusion.

**Supplementary Information:**

The online version contains supplementary material available at 10.1186/s13000-024-01519-9.

## Background

Waldenström’s macroglobulinemia (WM) is defined as a lymphoplasmacytic lymphoma (LPL) involving the bone marrow (BM) with presence of IgM monoclonal protein, and comprises > 95% of all LPL cases [1]. Extramedullary extension of WM/LPL is rare (4.4%), and the most common sites of invasion are the lung (30%), soft tissue (21%), cerebrospinal fluid (23%), kidney (8%), and bone (9%) [2]. Approximately 10% of WM/LPL cases are accompanied by amyloid immunoglobulin (Ig) light chain (AL) amyloidosis [3]. When these conditions coexist, overall survival is significantly worse than that for WM/LPL alone [4, 5]. Cardiac amyloidosis is a risk factor for sudden death [6], and, because AL amyloidosis frequently affects the heart [7], it is thought to be associated with unexpected sudden death among patients with WM/LPL.

Rituximab-based regimens have been predominant in the management of WM [3]. Infusion-related reactions (IRRs) are a primary concern with rituximab, although it is generally better tolerated with less toxicity than conventional anticancer agents [8]. The most common IRRs are mild to moderate and include fever, chills, rigors, skin rash, pruritus, nausea, and headache. More severe reactions may include hypotension, angioedema, hypoxia, and bronchospasm, as well as myocardial infarction, ventricular fibrillation, cardiogenic shock, and anaphylaxis [8].

Here, we present an autopsy case of an 84-year-old man who died suddenly after receiving the initial infusion of rituximab for WM/LPL.

## Case presentation

An 84-year-old man with clinically diagnosed diabetic nephropathy with microalbuminuria and Alzheimer’s disease presented to a general hospital for treatment of WM/LPL. The laboratory test and flow cytometry findings at the diagnosis of WM/LPL are summarized in Table 1. He scored highly on the revised International Prognostic Scoring System for WM (3: high) [9]. A pathology report for a BM aspirate indicated infiltration of lymphocytic cells with small-to-medium-sized nuclei that were positive for CD20 and CD79a, scattered CD138-positive cells, and higher abundance of IgM- and Igλ-positive cells than IgG-, IgA-, and Igκ-positive cells. Lymphocytes, including lymphocytic cells, accounted for 28.2% of all cells, and plasma cells for 3.4%. Chromosome examination of BM fluid revealed no specific abnormalities. *MYD88* and *CXCR4* testing was not performed. A computed tomography scan of the chest and abdomen showed bilateral pleural effusions and subcutaneous edema, with no splenomegaly or enlarged lymph nodes. No clinically evident cardiac dysfunction was present, and there were no risk factors for cardiovascular disease such as hypertension or obesity, with no family history of these conditions. Furthermore, there was no symptom suggestive of hyperviscosity syndrome. Because of progressive anemia observed in the clinical course, treatment with rituximab and ibrutinib was scheduled, and the first rituximab injection was performed on the day after the diagnosis. At 1 h after ingestion of ibuprofen (200 mg) and olopatadine hydrochloride (5 mg), the initial intravenous rituximab infusion was started with an infusion rate of 50 mg/h. Approximately 1 h after the initiation, he experienced respiratory disturbance, chills, and elevated blood pressure (180/102 mmHg), suggestive of IRRs. Thus, he received another oral administration of ibuprofen (200 mg) and olopatadine hydrochloride (5 mg). After 30 min, the chills resolved and the rituximab infusion resumed. Approximately 2 h after the initiation, his temperature increased to 38.6 °C, but he exhibited no additional symptoms and his vital signs remained stable. Consequently, the infusion rate was increased to 100 mg/h, with subsequent increments of 50 mg/h. Approximately 4 h after the initiation, intravenous administration of acetaminophen (500 mg) was performed because his temperature rose to 38.9 °C. Finally, the rituximab administration (500 mg) was completed in approximately 5 h. Ultimately, no steroids were administered. After the infusion, his temperature remained high (38.3 °C) and he complained of general weakness and inability to walk. Upon returning home, he was administered acetaminophen (300 mg), resulting in the resolution of his fever. However, he remained unable to eat. When his wife checked on him at 6 h after completion of the infusion, there appeared to be no issues. However, the next morning, he was found dead in his bedroom with postmortem changes. Ibrutinib was not taken prior to death because it was scheduled to be started on the morning of the day of death.

**Table 1:** Laboratory data at the diagnosis

Complete blood count				Chemistry^*^				Flow cytometry^**^		
WBC	5870	/µL		TP	8.7	g/dL		CD3	30.1	%
Basophiles	20	/µL		Alb	2.3	g/dL		CD4	10.0	%
Eosinophiles	10	/µL		T-Chol	92	mg/dL		CD5	20.4	%
Neutrophiles	4490	/µL		BUN	18.4	mg/dL		CD7	30.5	%
Lymphocytes	940	/µL		Cre	0.49	mg/dL		CD8	21.4	%
Monocytes	410	/µL		AST	10	U/L		CD10	12.7	%
RBC	2.96	x10^6^/µL		ALT	9	U/L		CD13	12.1	%
Hemoglobin	8.7	g/dL		γ-GT	13	U/L		CD14	1.4	%
HCT	28.9	%		ALP	73	U/L		CD19	58.2	%
Platelets	27.9	x10^4^/µL		LD	84	U/L		CD20	58.9	%
HbA1c	7.2	%		Na	138	mEq/L		CD21	0.6	%
Protein fractionation				K	4.6	mEq/L		CD23	2.7	%
Alb	32.6	%		Cl	102	mEq/L		CD24	37.1	%
*α*1	3.0	%		Glu	229	mg/dL		CD25	0.7	%
*α*2	6.6	%		CRP	4.85	mg/dL		CD38	28.8	%
*β*	6.1	%		IgG	1365	mg/dL		CD71	15.8	%
*γ*	51.7	%		IgA	64	mg/dL		SmIg-κ	2.0	%
A/G ratio	0.48			IgM	4638	mg/dL		SmIg-λ	54.9	%

Abbreviations: A/G albumin/globulin; ALP, alkaline phosphatase; ALT, alanine aminotransferase; AST, aspartate aminotransferase; BUN, blood urea nitrogen; Cre, creatinine; CRP, C-reactive protein; Glu, glucose; HCT, hematocrit; Ig, immunoglobulin; LDH, lactate dehydrogenase; RBC, red blood cells; SmIg, B cell surface immunoglobulin; T-Chol, total cholesterol; TP, total protein; WBC, white blood cell; γ-GT, γ-glutamyltransferase.

^*^ The level of serum β2-microglobulin was 3.4 mg/L in a laboratory test performed approximately 2 months prior to this examination.

^**^ Evaluated using bone marrow aspirate.

The estimated time of death was approximately 15 h after the end of the intravenous rituximab therapy. He was 163 cm tall and weighed 55 kg (body mass index: 20.7). Autopsy revealed 450 and 150 mL of clear yellow fluid in the right and left thoracic cavity, respectively. Toxicology testing was negative for ethanol and major drugs.

**Fig. 1:**
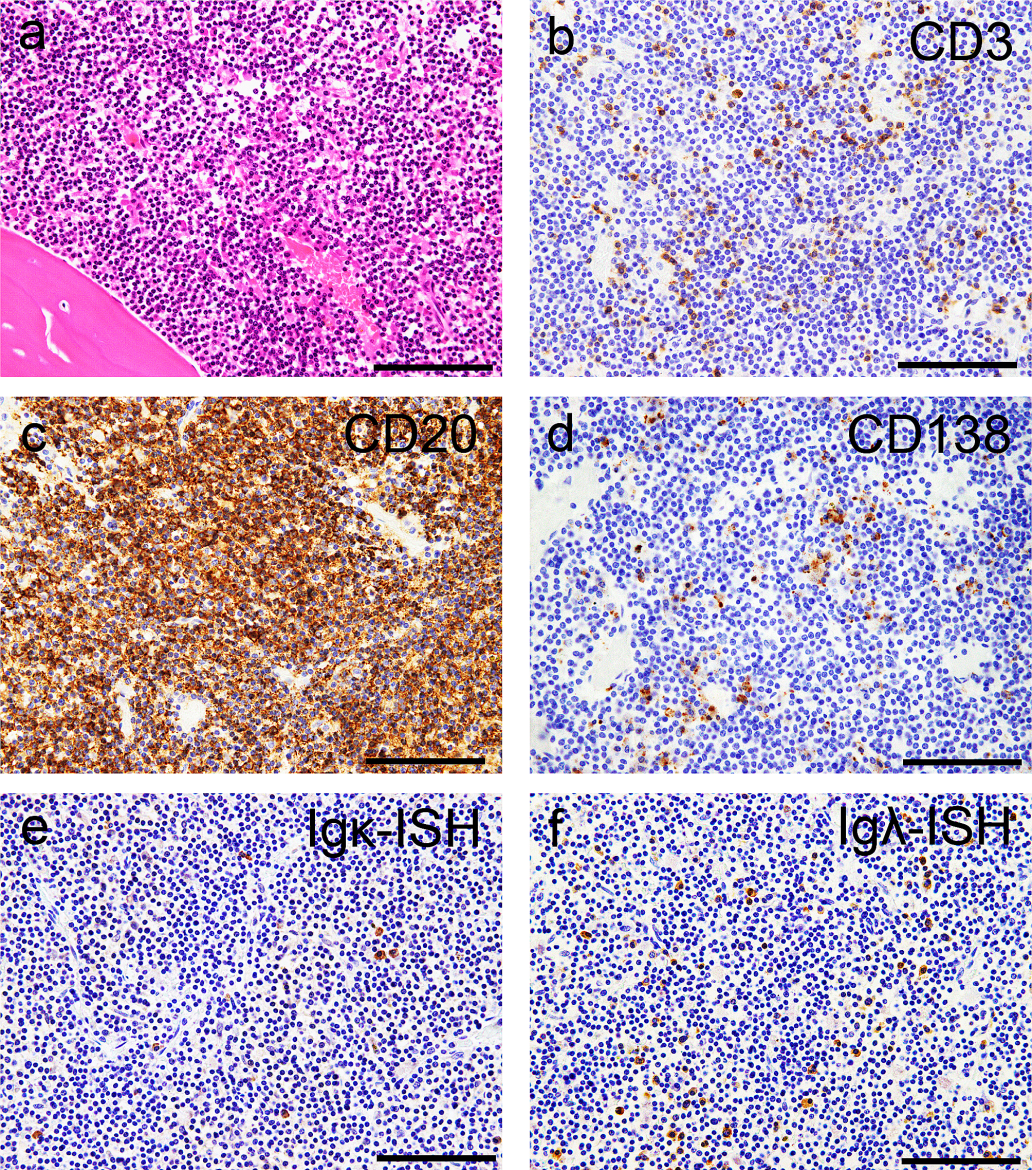
Representative microphotographs of the bone marrow. (**a**) Hematoxylin and eosin staining. (**b–d**) Immunohistochemistry for CD3 (**b**), CD20 (**c**), and CD138 (**d**). (**e**, **f**) In situ hybridization (ISH) for immunoglobulin κ light chain (Igκ) (**e**) and Igλ (**f**). (**a**) Infiltration of lymphocytic cells with small-to-medium-sized nuclei is observed (**a**). (**b**, **c**) The cells are focally positive for CD3 (**b**) and diffusely positive for CD20 (**c**). (**d**) Scattered CD138-immunoreactive cells are also noted. (**e**, **f**) Although Igλ-ISH-positive cells are observed more frequently than Igκ-ISH-positive cells, light chain restriction is indefinite in the autopsy materials. Scale bars: 100 μm

In the BM, histopathological evaluation revealed infiltration of lymphocytic cells with small-to-medium-sized nuclei (Fig. 1a). The cells were focally positive for CD3 (Fig. 1b) and diffusely positive for CD20 (Fig. 1c), with scattered CD138-positive cells observed in the foci (Fig. 1d). Light chain restriction was indefinite (Fig. 1e, f).

**Fig. 2:**
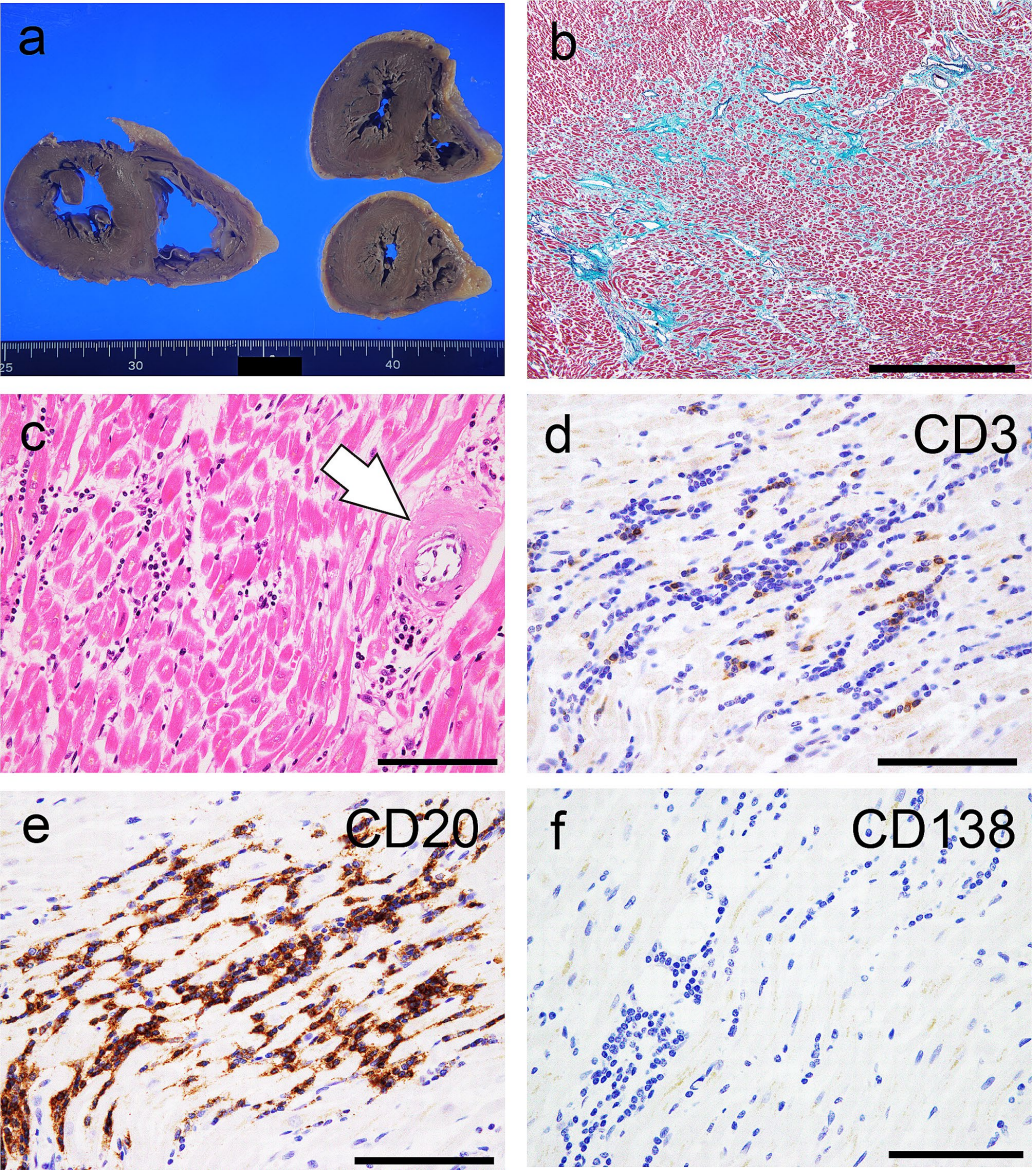
Representative macroscopic and microscopic photographs of the heart. (**a**) Macroscopic findings of the heart. (**b–f**) Elastica-Masson staining. (**c**) Hematoxylin and eosin staining. (**d–f**) Immunohistochemistry for CD3 (**d**), CD20 (**e**), and CD138 (**f**). (**a**) Mild left ventricular hypertrophy is noted. Discoloration of the myocardium is not evident. (**b**) Moderate fibrosis of the myocardium is observed. (**c**) At high magnification, infiltration of small-to-medium-sized lymphoid cells and eosinophilic deposition on the vessel (arrow) are observed. (**d–f**) The infiltrating lymphocytes are focally positive for CD3 (**d**) and diffusely positive for CD20 (**e**), while CD138-immunoreactive cells are scarce (**f**). Scale bars: 1 mm (**b**); 100 μm (**c–f**)

**Fig. 3:**
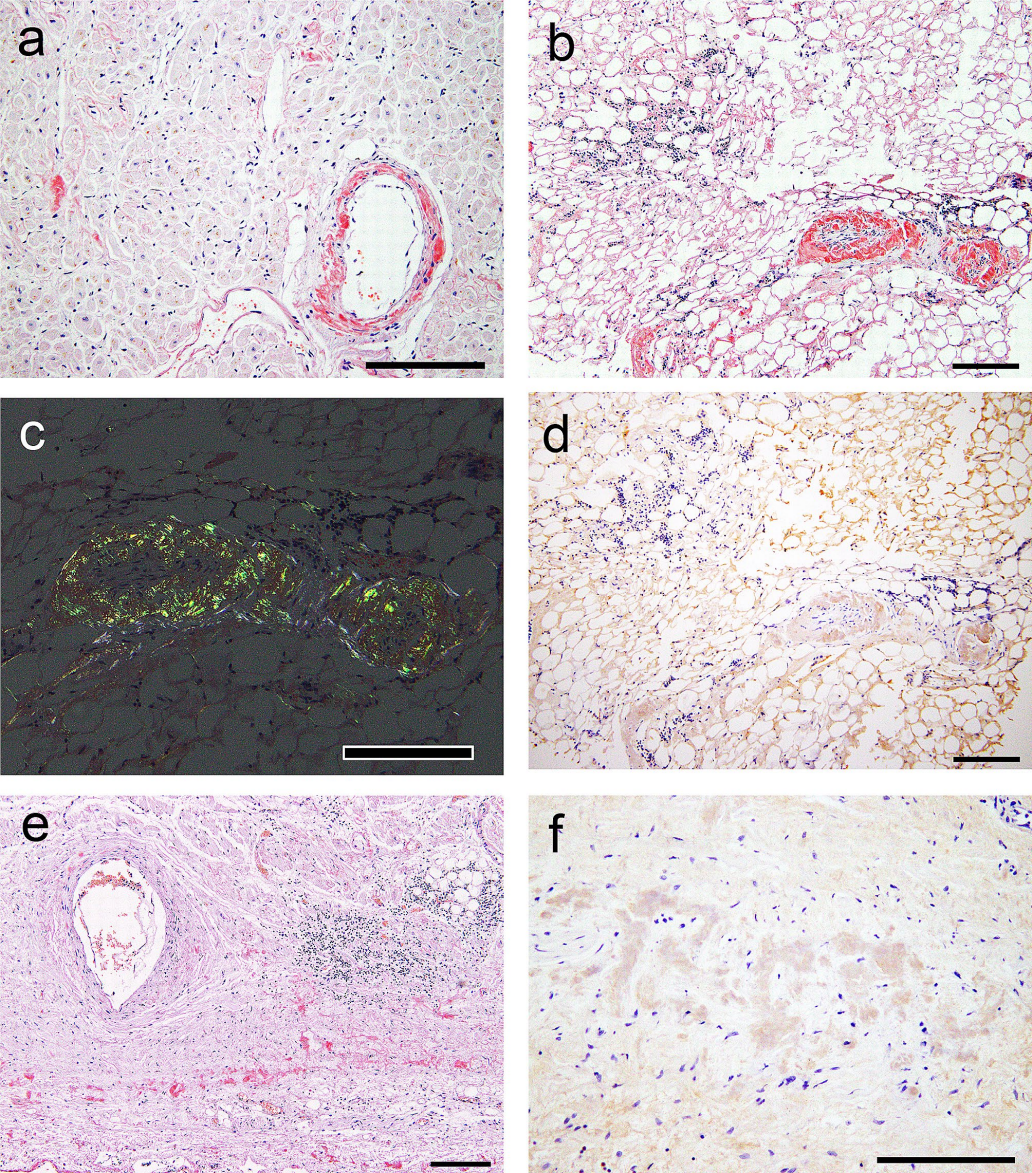
Representative microphotographs of the deposits in the heart. (**a–c**, **e**) Phenol Congo red (CR) staining under bright field observation (**a, b, e, f**) and polarized light observation (**c**). (**d, f**) Immunohistochemistry for Igλ. (**a**) Left ventricle. (**b–d**) Epicardium. (**e**, **f**) Sinoatrial node and surrounding atrial tissue. (**a**) CR-positive deposits are observed in the interstitium and vessels in the left ventricle. (**b**) More CR-positive deposits are observed in the epicardium than in the myocardium, and lymphoid cell infiltration is also observed. (**c**) The deposits exhibit apple-green birefringence under polarized light. (**d**) The amyloid deposits are positive for Igλ. (**e**, **f**) Amyloid deposits are observed in the interstitium of the sinoatrial node and show immunoreactivity for Igλ. Scale bars: 200 μm

**Fig. 4:**
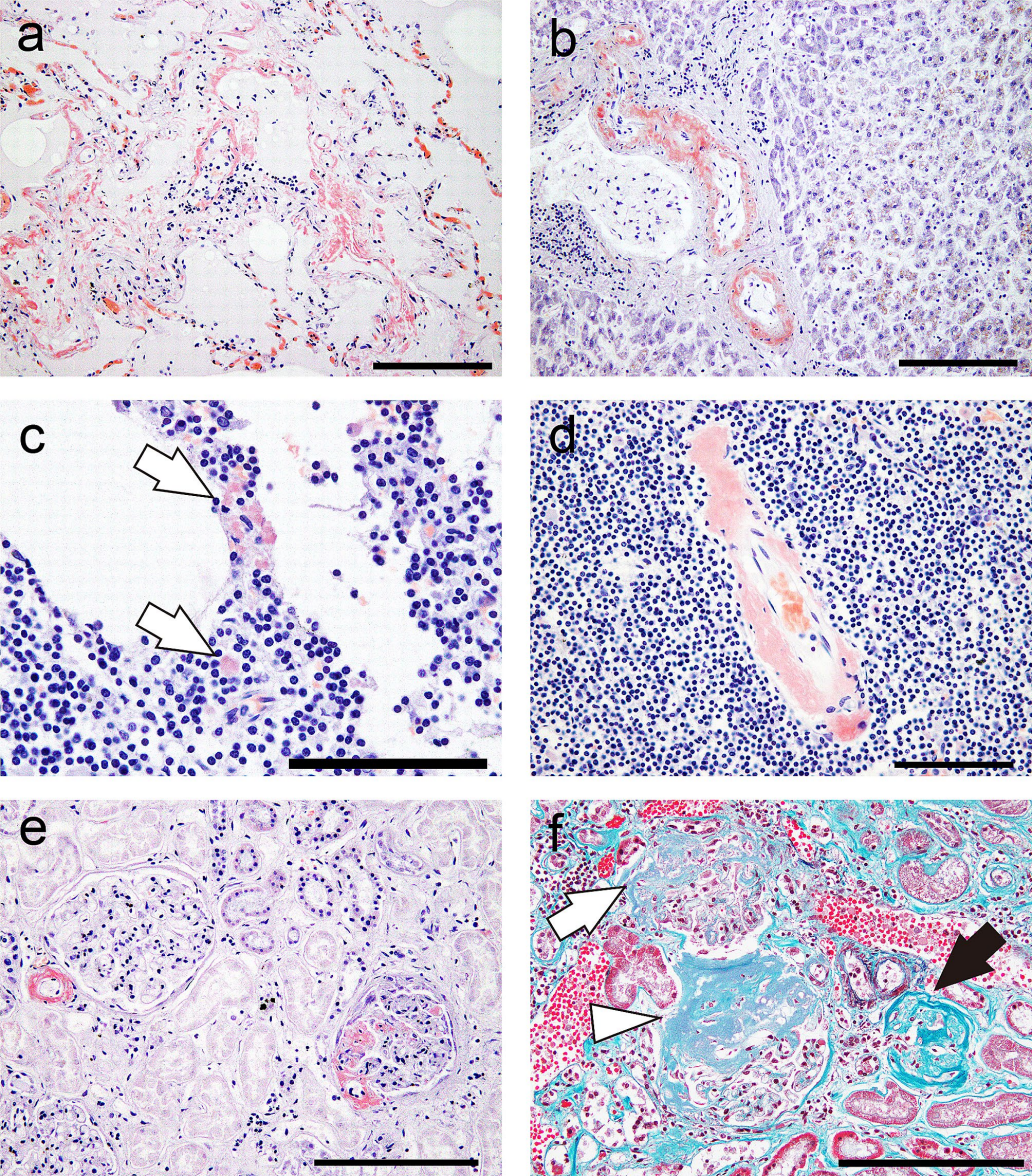
Representative micrographs of amyloid deposits in organs other than the heart. (**a–e**) Phenol Congo red staining. (**f**) Elastica-Masson staining. (**a**) Lung. (**b**) Liver. (**c, d**) Bone marrow. (**e, f**) Kidney. (**a–e**) Amyloid deposition is mainly observed on the vessels. (**c, d**) In the bone marrow, stromal (**c**, arrow) and vascular (**d**) amyloid deposition is noted. (**f**) In the kidney, a mixture of pathologies in the glomeruli, including amyloid deposition (white arrow), global sclerosis (black arrow), and amyloid deposition and sclerosis (arrowhead), is observed. Scale bars: 200 μm (**a, b, e, f**); 100 μm (**c, d**)

The heart weighed 393 g, and no obvious discoloration of the myocardium was observed (Fig. 2a). Histopathological evaluation revealed moderate interstitial fibrosis without significant myocardial necrosis (Fig. 2b). Infiltration of lymphocytic cells and eosinophilic deposition were observed (Fig. 2c). On immunohistochemical staining, the infiltrating cells were predominantly positive for CD20 (Fig. 2d–f), indicating the involvement of LPL. The deposits in the ventricles and epicardium showed strong congophilia and apple-green birefringence under polarized light (Fig. 3a–c), confirming amyloid deposition. The amyloid deposits were immunoreactive for Igλ (Fig. 3d) and negative for Igκ and prealbumin. These findings were verified by consultation with the Group of Surveys and Research of Amyloidosis in Japan (Supplementary Figure S1) [10]. ALλ deposition was identified in the sinoatrial node (Fig. 3e, f). A moderate amount of amyloid atrial natriuretic factor deposition was observed in the atrial septum (Supplementary Figure S2). ALλ deposition was found in all organs examined except for the brain (Fig. 4a–e). Amyloid deposition in the stroma and vessels within the BM, a hallmark of AL amyloidosis [11, 12], was noted (Fig. 4d). In the kidney, amyloid deposition and glomerular sclerosis were evident (Fig. 4e, f).

The right and left lungs weighed 866 and 692 g, respectively, and exhibited severe congestive edema. The liver weighed 1415 g, and had severe congestion. Histopathological examination revealed severe edema in both lungs and severe congestion and centrilobular-type necrosis in the liver, suggesting the occurrence of severe circulatory failure.

The brain weighed 1327 g and showed general atrophy. Neuropathological examination revealed edematous change and an intermediate level of Alzheimer’s disease-related pathology based on the National Institution on Aging-Alzheimer’s Association guideline (A3B2C3) (Supplementary Figure S3) [13]. The immunohistochemical and in situ hybridization techniques employed in this case are summarized in Supplementary Table S1.

Laboratory tests on postmortem serum samples revealed elevation of interleukin (IL)-1β (34 pg/mL [reference range: ≤10 pg/mL]), IL-6 (176 pg/mL [< 7.0 pg/mL]), tumor necrosis factor-α (1.95 pg/mL [0.75–1.66 pg/mL]), and soluble IL-2 receptor (sIL-2R) (4980 U/mL [157–474 U/mL]). No light chain restriction was confirmed (free Igκ: 30.4 mg/L [3.3–19.4 mg/L]; free Igλ: 52.0 mg/L [5.7–26.3 mg/L]; κ/λ ratio: 0.58 [0.26–1.65]), and the presence of IgM-λ monoclonal protein was noted (Supplementary Figure S4).

## Discussion and conclusions

In the present case, IRRs associated with the initial rituximab infusion triggered rapidly progressive multiple organ failure, ultimately leading to death. Although laboratory test findings revealed increased inflammatory cytokine levels consistent with IRRs, the indication for initiating rituximab therapy (progressive anemia) [3], the total dose administered, and the infusion rate were all appropriate, along with the prophylactic use of antiallergic medications. Meanwhile, the patient had a diminished systemic organ reserve capacity due to certain pathological conditions, including WM/LPL exhibiting cardiac involvement accompanied by clinically undiagnosed systemic ALλ amyloidosis, diabetes, and aging-related pathologies. Collectively, it was considered that predicting and preventing death due to IRRs may have been very challenging in this case.

IRRs are common side effects of rituximab therapy. These reactions were reported to occur most frequently during the first administration of rituximab in patients with B-cell non-Hodgkin lymphoma (72–87%) [14]. However, the vast majority of the IRRs are grade 1 or 2 severity, based on the National Cancer Institute Common Terminology Criteria for Adverse Events, Grade 3 and 4 reactions are uncommon, and mortality associated with administration of rituximab is extremely rare (0.04–0.07%) [14, 15]. Tachi et al. [16] identified sIL-2R > 2000 U/L, hemoglobin < 13.0 g/dL (for men), and absence of steroid premedication as risk factors for development of IRRs after rituximab administration in patients with B-cell non-Hodgkin lymphoma within a Japanese cohort. Because the present patient was elderly and had all of these risk factors, he is presumed to have had a high likelihood of developing severe IRRs.

IRRs are immune manifestations that can arise through different mechanisms, including IgE-mediated hypersensitivity, anaphylactoid reaction (IgE-independent allergic reaction), immunogenicity of rituximab, complement activation, and cytokine-release syndrome [8]. In the present case, rituximab was administered for the first time, and thus involvement of IgE-mediated mechanisms was considered unlikely. However, the elevated levels of inflammatory cytokines indicate that cytokine-release syndrome was one of the predominant contributors to the IRRs in this case. Nevertheless, it should be noted that patients with WM/LPL were reported to have higher IL-6 levels at diagnosis than normal control subjects [17]. Thus, it remains unclear whether these cytokine levels were elevated by the tumor itself or by the administration of rituximab. Further accumulation of cases is needed to improve the predictive accuracy for patients who may experience severe IRRs. Also, in this case, there was almost no tissue damage due to inflammatory cell infiltration in any organs, suggesting that tissue damage may not be directly related to the deterioration of the general condition in acute phase IRRs.

The combination of AL amyloid deposition and WM/LPL infiltration into the heart was observed in the present case. There are no detailed data on the frequency of LPL involvement based on histological examination of the heart. However, considering the prevalence of AL amyloidosis in WM/LPL (approximately 10%) [3] and the occurrence of extramedullary extension in WM/LPL (approximately 4%) [2], the coexistence of both conditions is considered extremely rare. To the best of our knowledge, there is only one documented case with histological evidence confirming the simultaneous presence of these two conditions [1]. Because the quantity of infiltrating LPL cells and deposited amyloid was not substantial, it is unlikely to have significantly impacted cardiac function, consistent with the absence of obvious cardiac symptoms in the clinical course. However, the combination of these conditions could serve as a potential risk factor for the rapid deterioration of cardiac function in the event of IRRs.

In conclusion, we have presented for the first time an autopsy case of sudden unexpected death attributed to IRRs following the initial administration of rituximab for WM/LPL. While no significant inflammatory cell infiltration was observed in any organs, LPL infiltration and amyloid deposition in the heart, the presence of diabetes, age-related pathologies, elevated sIL-2R, and reduced hemoglobin, along with the absence of prior prophylactic steroid administration, were identified as potential contributors to the development of acute IRRs in this elderly patient. The present findings suggest that older patients with high-risk WM/LPL who exhibit severe IRRs during the first administration of rituximab should be hospitalized and closely monitored until their symptoms are completely resolved, in addition to investigating potential complications of WM/LPL before infusion. Furthermore, this study suggests that, even in the absence of obvious clinical symptoms, it is important to conduct a detailed examination of cardiac function and assess for complications of AL amyloidosis before initiating treatment for WM/LPL.

### Electronic supplementary material

Below is the link to the electronic supplementary material.

Supplementary Material 1

## Data Availability

No datasets were generated or analysed during the current study.

## Abbreviations

ALλ: immunoglobulin λ-light chain-derived amyloid
BM: bone marrow
IPPs: Infusion-related reactions
LPL: lymphoplasmacytic lymphoma
WM: Waldenström’s macroglobulinemia
