# Supplementary material for: Sudden unexpected death after initial infusion of rituximab for Waldenström macroglobulinemia/lymphoplasmacytic lymphoma: an autopsy case
Source: Diagn Pathol. 2024 Jun 28;19:89. doi: 10.1186/s13000-024-01519-9 (PMC11212197; doi:10.1186/s13000-024-01519-9)
Supplement: Supplementary file 1 — Supplementary Material 1 [file 13000_2024_1519_MOESM1_ESM.docx]

**Online Supplementary Material**

Sudden death due to infusion-related reactions in an elderly man after initial infusion of rituximab for Waldenström macroglobulinemia/lymphoplasmacytic lymphoma: an autopsy case

Shojiro Ichimata^1^, Yukiko Hata^1^, Kazuhiro Nomoto^2^, Tsutomu Sato^3^, Naoki Nishida^1^

^1^Department of Legal Medicine, Faculty of Medicine, University of Toyama, Toyama, Japan

^2^Department of Pathology, Koseiren Takaoka Hospital, Takaoka, Japan

3Department of Hematology, Toyama University Hospital, Toyama, Japan.

**List of Supplementary Materials**

Supplementary Tables: 1

Supplementary Figures: 4

**Supplementary Table S1.** Summary of the antibodies and *in situ* hybridization probes used in the study

| **Antibody** | **Source** | **Clone** | **Dilution** | **Antigen retrieval** |
| --- | --- | --- | --- | --- |
| CD3 | Dako | F7.2.83 | 1:200 | Heat (pH9, 20 min) |
| CD20 | Novocastra Vector Lab | L26 | 1:100 | Heat (pH9, 20 min) |
| CD138 | Agilent | MI15 | 1:200 | Heat (pH9, 20 min) |
| Igλ (IHC) | Abcam | polyclonal | 1:1000 | 98% FA (1 min) |
| Igκ (IHC) | DB Biotech | H16-E | 1:500 | 98% FA (1 min) |
| Prealbumin | Abcam | EPR3219 | 1:2000 | 98% FA (1 min) |
| ANF | GeneTex | 23/1 | 1:20000 | 98% FA (1 min) |
| Phosphorylated tau | Endogen | AT8 | 1:1000 | Heat (pH9, 20 min) |
| Amyloid-β | Dako | 6F/3D | 1:50 | 98% FA (10 min) |
| **ISH probe** | **Source** | **Catalog #** | **Dilution** | **Pretreatment** |
| Igκ (ISH) | Leica Biosystems | PB0645 | Ready to use | Enzyme 1 (10 min) |
| Igλ (ISH) | Leica Biosystems | PB0669 | Ready to use | Enzyme 1 (10 min) |

**Abbreviations:** ANF, atrial natriuretic factor; FA, formic acid; Ig, immunoglobulin; ISH, *in situ* hybridization.

Immunostaining and ISH were performed using Leica Bond-IV automation and Leica Refine detection kits (Leica Biosystems, Bannockburn, IL, USA) in accordance with the manufacturer’s instructions. All sections were counterstained with hematoxylin.

**
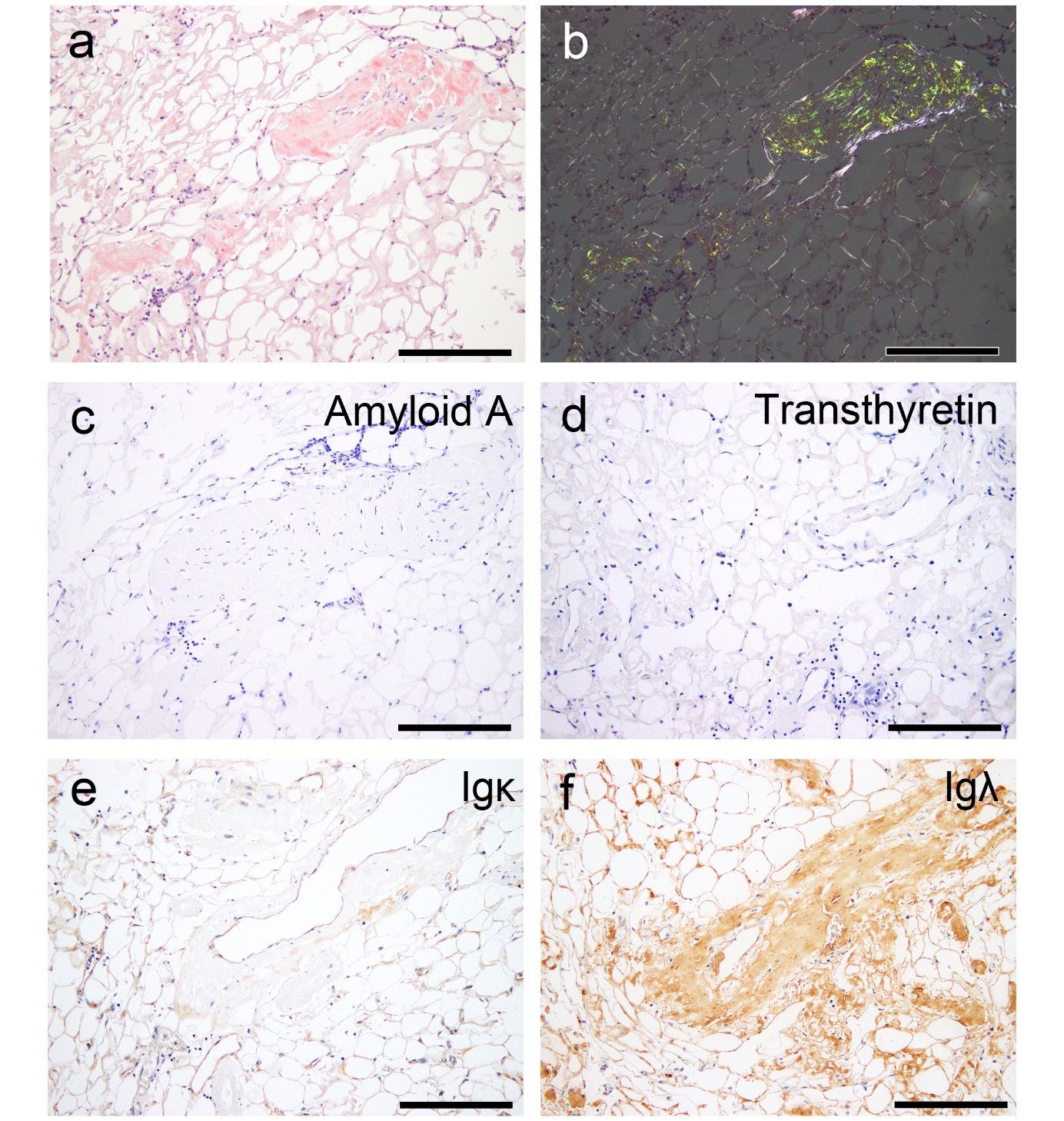
Supplementary Figure S1.** Representative micrographs of the Congo-red staining and immunohistochemistry in the epicardium.

(a, b) Congo red staining under bright field (a) and polarized light (b) observation. (c–f) Immunohistochemistry for amyloid A (c), transthyretin (d), immunoglobulin (Ig) κ-light chain (Igκ) (e) and Igλ (f). Amyloid deposits are positive for Igλ (f) and negative for other antibodies. Scale bars: 200 μm.

**
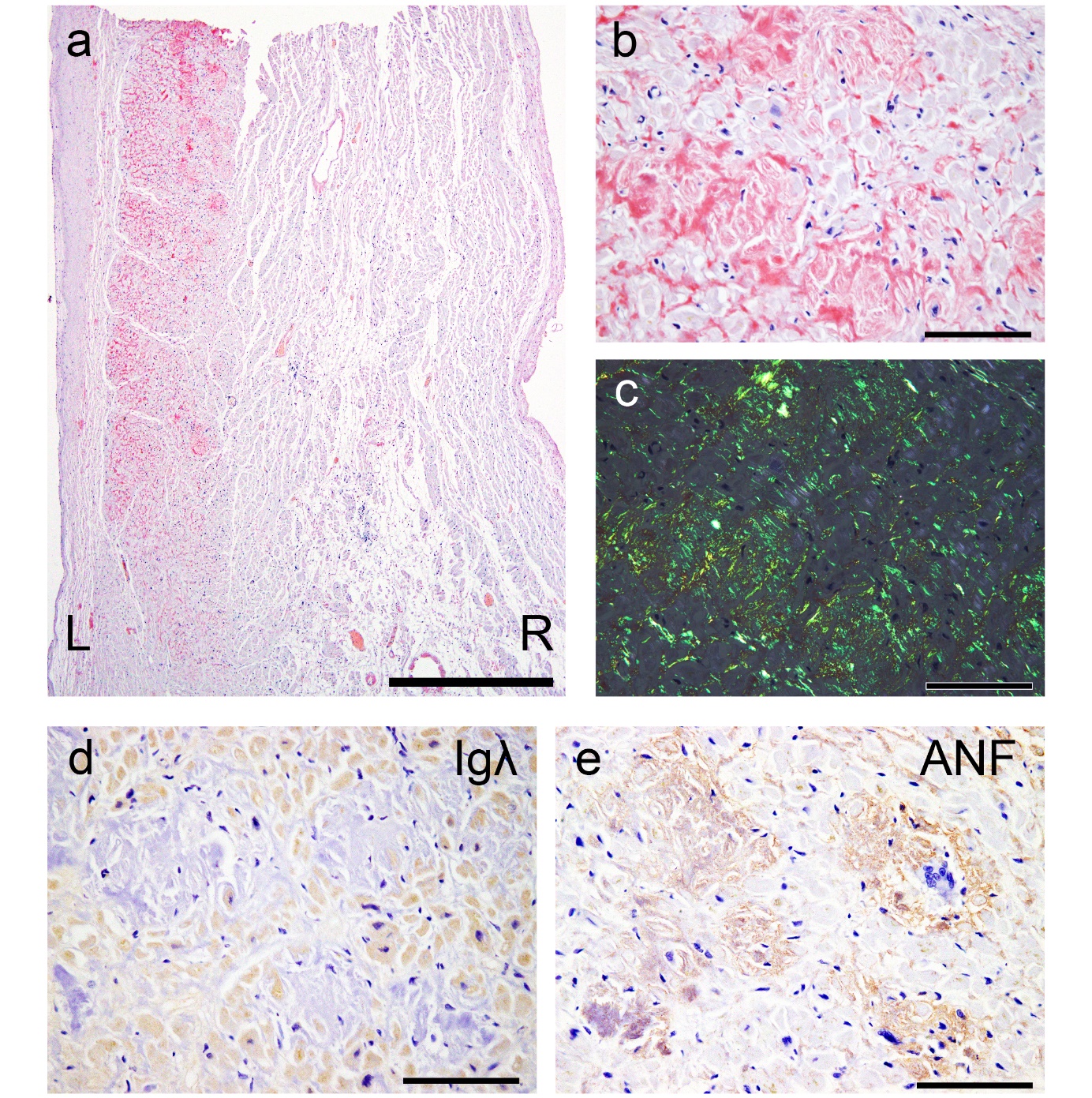
Supplementary Figure S2.** Representative microphotographs in the atrial septum.

(a–c) Phenol Congo red staining under bright field (a, b) and polarized light observation (c). (d, e) Immunohistochemistry for Igλ (d) and ANF (e). (a) The left (L) side of the atrial septum showed strong congophilia compared with the right (R) side. (b, c) The deposits show typical apple-green birefringence under polarized light observation. (d, e) Immunohistochemically, the amyloid deposits are negative for Igλ (d) and positive for ANF (e). Scale bars: 1 mm (a); 100 μm (b–e).


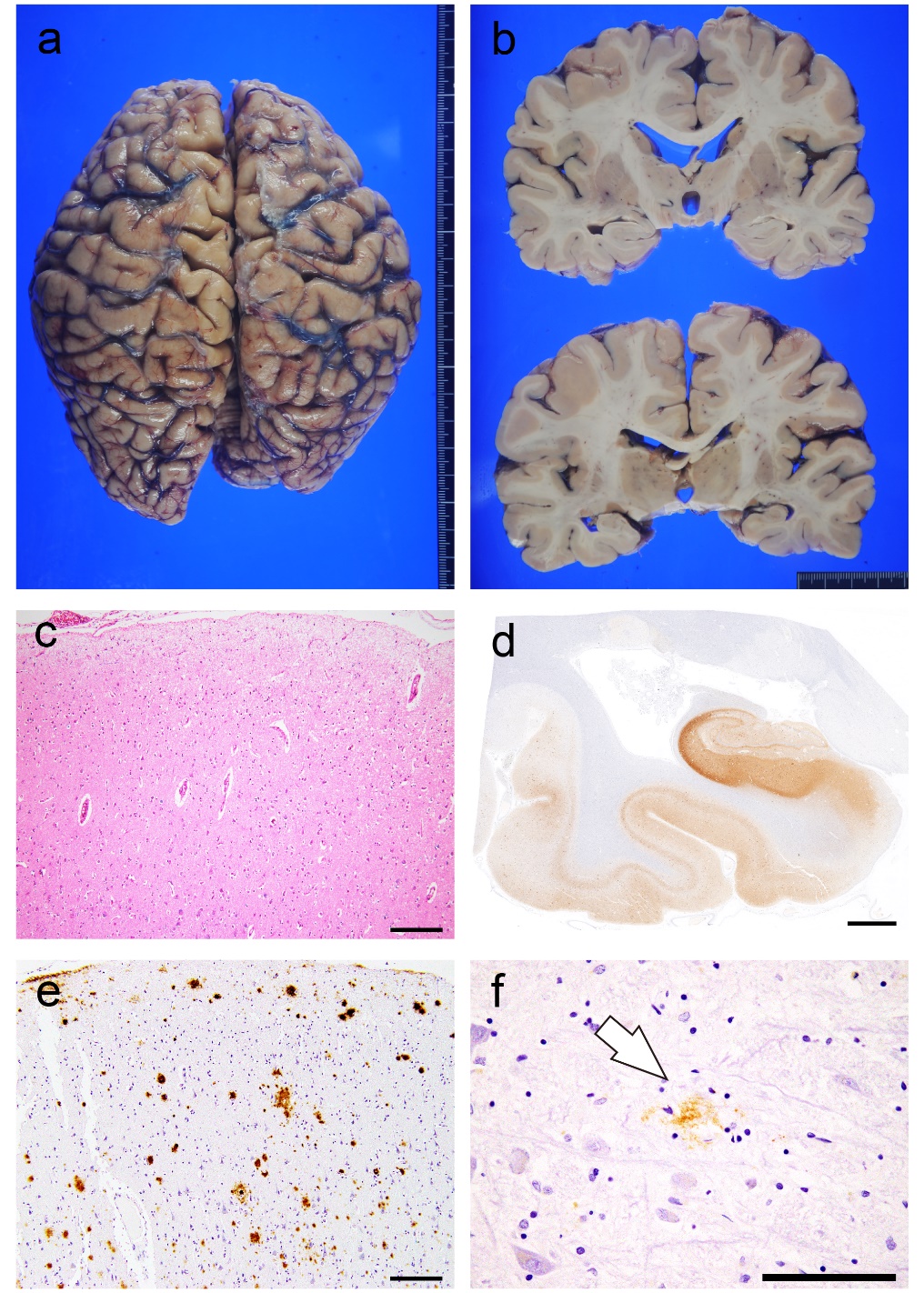
**Supplementary Figure S3.** Representative microphotographs of the brain.

(a, b) Macroscopic examination of the brain. (c) Luxol fast blue-hematoxylin and eosin staining. (d–f) Immunohistochemistry for phosphorylated tau (d) and amyloid-β (e, f). (c, e) Cerebral cortex (frontal lobe). (d) Hippocampus to inferior temporal gyrus. (f) Medulla oblongata. (a, b) Macroscopically, the brain exhibits overall atrophy. (c) Neuropathologically, enlargement of the perivascular space and edema of the interstitium are noted. (d) Phosphorylated tau-immunoreactive neurofibrillary tangles are observed up to the inferior temporal gyrus. (e, f) Amyloid-β is diffusely observed in the cerebral cortex (e), and its deposition extends into the medulla oblongata (f, arrow). Scale bars: 3 mm (d); 200 μm (c, e); 100 μm (f).

**Supplementary Figure S4.** Representative findings in the immune-electrophoresis (a) and immunofixation electrophoresis analysis (b).


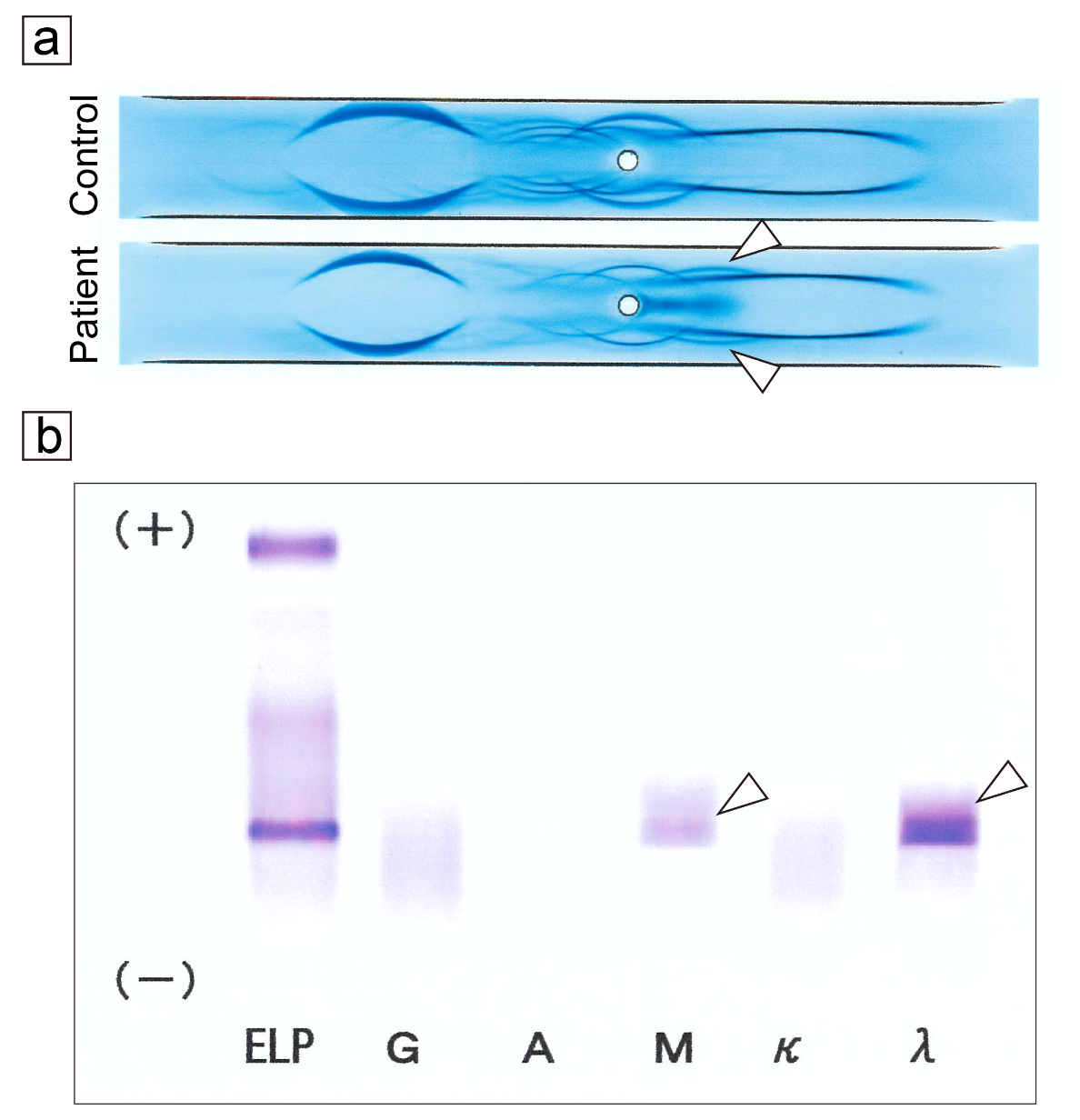


(a) Reductions are noted in albumin, prealbumin, α2-macroglobulin, transferrin, and β1c/β1a-globulin (C3). Regarding immunoglobulins, IgA is diminished, IgG is within the normal range, and IgM is elevated with monoclonal-like changes (arrowheads). (b) IgM-λ monoclonal protein is observed (arrowhead). Abbreviations: ELP, serum protein electrophoresis (protein fractionation); G, anti-IgG antibody; A, anti-IgA antibody; M, anti-IgM antibody; κ, anti-Igκ antibody; λ, anti-Igλ antibody.
